# Socioeconomic status and duration and pattern of sickness absence. A 1-year follow-up study of 2331 hospital employees

**DOI:** 10.1186/1471-2458-10-643

**Published:** 2010-10-25

**Authors:** Trine R Kristensen, Signe M Jensen, Svend Kreiner, Sigurd Mikkelsen

**Affiliations:** 1 Department of Occupational and Environmental Medicine, Copenhagen University Hospital, Bispebjerg, Bispebjerg Bakke, DK-2450 Copenhagen NV, Denmark; 2 Department of Biostatistics, University of Copenhagen, Oster Farimagsgade 5,B, DK-1014 Copenhagen K, Denmark

## Abstract

**Background:**

Sickness absence increases with lower socioeconomic status. However, it is not well known how this relation depends on specific aspects of sickness absence or the degree to which socioeconomic differences in sickness absence may be explained by other factors.

The purpose of the study was to examine differences in sickness absence among occupational groups in a large general hospital; how they depend on combinations of frequency and duration of sickness absence spells; and if they could be explained by self-reported general health, personal factors and work factors.

**Methods:**

The design is a 1-year prospective cohort study of 2331 hospital employees. Baseline information include job title, work unit, perceived general health, work factors and personal factors recorded from hospital administrative files or by questionnaire (response rate 84%). Sickness absence during follow-up was divided into short (1-3 days), medium (4-14 days) and long (>14 days) spells, and into no absence, "normal" absence (1-3 absences of certain durations) and "abnormal" absence (any other absence than "normal"). Socioeconomic status was assessed by job titles grouped in six occupational groups by level of education (from doctors to cleaners/porters). Effects of occupational group on sickness absence were adjusted for significant effects of age, gender, general health, personal factors and work factors. We used Poisson or logistic regression analysis to estimate the effects of model covariates (rate ratios (RR) or odds ratios (OR)) and their 95% confidence intervals (CI).

**Results:**

With a few exceptions sickness absence increased with decreasing socioeconomic status. However, the social gradient was quite different for different types of sickness absence. The gradient was strong for medium spells and "abnormal" absence, and weak for all spells, short spells, long spells and "normal" absence. For cleaners compared to doctors the adjusted risk estimates increased 4.2 (95% CI 2.8-6.2) and 7.4 (95% CI 3.3-16) times for medium spells and "abnormal" absence, respectively, while the similar changes varied from 0.79 to 2.8 for the other absence outcomes. General health explained some of the social gradient. Work factors and personal factors did not.

**Conclusions:**

The social gradient in sickness absence was different for absences of different duration and patterns. It was strongest for absences of medium length and "abnormal" absence. The social gradient was not explained by other factors.

## Background

Several studies show that sickness absence increases with decreasing socioeconomic status [1-18]. Most studies use a single sickness absence modality as outcome, e.g. number of absence days, any absence spell, or absence spells of a certain duration [5,8,11-17]. However, sickness absence is a complex phenomenon and it's causes vary with frequency and duration of absence spells [4,19]. The impact of socioeconomic status on different aspects of sickness absence could also vary due to socioeconomic differences in work conditions, cultural background, personal factors and health. Only a few studies have examined this problem and most of them consider only a dichotomy of short and long spells [1,3,4,6,7]. We found only two studies that report associations between socioeconomic status and incidence of sickness absence spells divided into more than two duration categories [2,10]. Only a minority have no sickness absence during a calendar year, but they always serve as the "normal" reference group. However, a few absences per year is quite normal and could be independent of work factors, personal factors, socioeconomic status or other explaining factors, eg. a flue or a broken leg. The "pattern" of different combinations of frequency and duration of absence spells and "normal" versus "abnormal" sickness absence would seem to be a natural field of sickness absence research, including effects of socioeconomic status and other factors. However, we found no studies dealing with these aspects of sickness absence.

Socioeconomic differences in sickness absence are of special interest if they can be explained. Health and working conditions vary with socioeconomic status [20,21] and predict sickness absence [22-24], and could therefore explain some of the socioeconomic differences in sickness absence. This was the case in several studies, although to a varying degree [1,5,8,10,17,18]. Just as socioeconomic effects on sickness absence may differ by different absence modalities (e.g. duration or frequency) the effects of other risk factors like health and working conditions could also vary with such differences.

In the present 1-year follow-up study of employees in a large general hospital we examined the relation between socioeconomic status and objectively recorded sickness absence divided into lengths of 1-3 days, 4-14 days and more than 14 days. Data were analysed as incidence rates and for those with any absence as odds of long versus short absences. We further studied the incidence of a specific sickness absence pattern labelled as "normal" and "abnormal". In the analyses, we adjusted for a large number of potential confounders or mediators to examine if they could explain the effects of socioeconomic status on sickness absence.

## Methods

The study population consisted of all employees at a general hospital in the county of Copenhagen, including somatic and psychiatric departments and supporting staff. Heads of departments were excluded for reasons of confidentiality because information on department and job title would reveal their identity. A baseline questionnaire about working conditions, health and personal circumstances was distributed to 3199 employees by departments and work units at the end of October 2000 followed by two reminders. 2687 (84%) questionnaires were returned before January 2001. By January 1^st ^2001, 148 employees had stopped working at the hospital and 123 did not work in the same work unit as when they answered the questionnaire. Thirteen had invalid employment data and 14 had invalid data on sickness absence. They were all excluded together with a small group of 58 employees, mainly workmen, with job titles that did not fit into our occupational groups, see below. The material consists of the remaining 2331 questionnaire responders. The participants worked in 28 departments divided into a total of 182 work units, comprising from 1 to 53 persons, the median being 11 persons. The work units were the lowest organisational level of the hospital, typically a ward or ambulatory.

The study was performed in the context of a political quest to improve working conditions and reduce sickness absence, and the purpose of the study was to supply the hospital and the departments with aggregated systematic information about perceived work conditions, health and sickness absence data. The study was supported by management and employee representatives. Participation was voluntary and only research staff had access to person-related data. This was all explained in information leaflets and in an introductory letter with the questionnaire. The study was reported to The Danish Data Protection Agency. According to Danish law, research projects based only on questionnaires do not need permission from an ethics committee.

### Sickness Absence

Participants were followed through hospital administrative data files from January 1^st ^2001 until the last date employed in the same working unit or to the end of 2001 whichever came first. Data on absences due to ordinary sickness absence was recorded by frequency and duration categories, including number of sickness absence days within each category. Pregnancy related sickness absence was excluded since we assumed it could have other risk factors than ordinary sickness absence.The records did not contain information on diagnoses. Part time sickness absence was used very seldom and such data were not available in this study.

Days at risk for starting a new spell of sickness absence was calculated as calendar days in the follow-up period , excluding Saturdays, Sundays and other holidays, days on vacation, and days of absence due to ordinary sickness, maternity leave, pregnancy related sickness or care of sick child. One day for each sickness absence spell was added since the first day of an absence spell starts as a day at risk.

We defined short spells of sick leave as 1-3 days, medium spells as 4-14 days and long spells as more than 14 days, based on administratively defined cut points in the aggregated absence data we had access to. The incidence rate was defined as all new sickness absence spells during the follow-up period divided by the risk time in the same period. We further grouped the respondents into two groups, one with a "normal" and the other with an "abnormal" absence pattern. Among persons with any absences, "normal" absence was defined as having no more than two short, one medium and one long spell, and altogether no more than three spells of any length during the observation period. Any other combination of absences was considered as "abnormal" absence. These pattern definitions are discussed below.

In Denmark a medical certificate is not mandatory for sickness absence spells but the employer may require one for absences >3 days. Employees can obtain compensation for up to one year of sickness absence. Mostly, and especially in higher occupational grades, the compensation is equal to the normal salary.

### Occupational group and socioeconomic status

Based on job titles from the hospital register, education and similarity of work content, we divided the hospital personnel into the following 6 occupational groups: 1) doctors, dentists, psychologists and other academic staff, 2) physiotherapists, midwives, medical laboratory technologists, social workers and alike, 3) nurses, 4) medical secretaries, office, and administrative workers, 5) nursing assistants, 6) cleaning personal, hospital porters, and various assistants. In the text we will refer to this ordered occupational grouping as a measure of graded socioeconomic status, although we acknowledge that there is no clear socioeconomic status difference between groups 2) and 3).

### Demographic and personal variables

Age and gender were registered in the hospital records. Information on cohabitation and children at home was gathered by questionnaire. Social support from family or friends was measured by a single item (If you have problems, can you obtain the help and support you need from your family and friends? (always, almost always, usually, often, now and again, rarely/never)) and personality characteristics was measured by three single items, covering negative affectivity (Do you as a person have a tendency to worry, or be nervous or a little pessimistic? (not at all, slightly, a little, some, quite a lot, fairly much, very much)), type A behaviour (Do you as a person have a tendency to be competitive, proud, ambitious and a little impatient? (same response alternatives)), and self efficacy (Are you the kind of person who can almost always solve difficult problems, cope with unforeseen situations and achieve your goals? (not at all, slightly, a little, some, quite a lot, fairly much, very much)). General health was measured by a single item from SF36 [25].

### Work time and schedule variables

Regular working hours per week, frequency of duties on evenings/nights, frequency of weekend duties, and overtime work was recorded by questionnaire.

### Work related psychosocial variables

Work related quantitative demands (4 items), cognitive demands (4 items) and emotional demands (3 items), decision authority (5 items) and skill discretion (4 items), support from colleagues and superiors at work (4 items), meaning of work (2 items), commitment to the workplace (4 items), predictability (2 items), sense of community (3 items), role-clarity (4 items), quality of leadership (5 items), and role-conflicts (1 item) were measured with scales and items from the first edition of the Copenhagen Psychosocial Questionnaire, COPSOQ [26]. An overall job demand scale was constructed by taking the mean of the 3 demand scales, and a control scale was constructed as the mean of the decision authority and skill discretion scales. Threats and violence was measured with a 3 item scale (Have you, within the last 12 months, during work been exposed to 1) verbal or written menaces?, 2) menacing behaviour?, 3) pushes, beating, kicks, bites? (response categories: no, yes once, yes 2-5 times, yes 5-10 times, yes >10 times)). We further used single items to measure overall job satisfaction (How satisfied are you with your work, all in all?, very satisfied, quite satisfied, satisfied, slightly dissatisfied, quite dissatisfied , very dissatisfied)), feeling like going to work (How much do you normally feel like going to work? (very much, reasonably much, to some extent, slightly reluctant, very reluctant, extremely reluctant)), feeling unsafe at work (Do you ever feel unsafe at work? (always, often , sometimes, never)), and a measure of available time and ressources which we interprete as a proxy of the effort required to perform the work tasks (Do you have sufficient time and resources to perform your tasks satisfactorily? (to a very great extent, to a great extent, to some extent, to a lesser extent, to a very little extent, hardly at all)), and reward (Do you feel your work efforts are sufficiently appreciated? (same response categories)). The proxy effort and reward items were included in the analyses by the ratio effort/reward [27]. In this set of variables we also included a single item to assess the overall degree of physical work demands (Is your work physically demanding? (to a very high degree, to a high degree, somewhat, to a low degree, to a very low degree)).

### Statistical analysis

The association between occupational group and number of incident sickness absence spells was examined in Poisson regression models allowing for overdispersion and with the logarithm of days at risk as offset. Rate ratios (RR) and their 95% confidence intervals (CI) for occupational groups with the group of doctors as reference were calculated for short, medium, long and any sickness absence spells. The associations between occupational group and "normal" and "abnormal" absence versus no absences were examined in logistic regression analyses with days at risk included as a covariate.

Among participants with absence spells we further examined the odds of a longer compared to a shorter sickness absence period. We defined three mutually exclusive groups of participants with sickness absence: 1) participants who had only had short absences, 2) participants with any medium but no long absences, and 3) participants with any long absences. In three separate analyses we examined the odds of belonging to one of these groups versus belonging to one of the others, excluding the third group The binary outcome was scored 1 for the longer and 0 for the shorter absence.

Odds ratios (OR) and their 95% CI for occupational groups with the group of doctors as reference were calculated.

Persons working in the same units might have unknown factors in common, factors that made them choose to work in the unit and factors due to influences from working in the unit. We included a random work unit effect in all regression analyses to adjust for these contextual similarities within work units [28].

The analyses were carried out stepwise, starting with an "empty" model including only the random work unit effect. Subsequent models all included occupational group, gender and age as explaining variables in addition to the work unit random effect. When analysing the incident number of short, medium and long absences, the presence (yes/no) of any other length of absence was also included among these covariates to control for the overlap between spells of different lengths. Groups of covariates were then introduced separately to see whether the covariates in the group could explain occupational group differences in sickness absence. The groups of covariates were: 1) work related psychosocial variables, 2) work time and schedule variables 3) personal variables and 4) general health. The factors included as covariates were considered to be potential risk factors for sickness absence [19] and could therefore act as mediators or confounders of the relation between socioeconomic status and sickness absence. A fully adjusted model including all covariates was reduced by backward elimination of non-significant (p > 0.05) covariates from these four groups, successively eliminating the least significant covariate (p > 0.05). The resulting models were controlled by re-introducing each of the eliminated covariates, one by one, and if significant (p ≤ 0.05), the covariate was retained in the model. We examined for interactions between occupational group and gender in all models. Analyses were made with PROC GLIMMIX, SAS (9.1).

## Results

**Figure 1:**
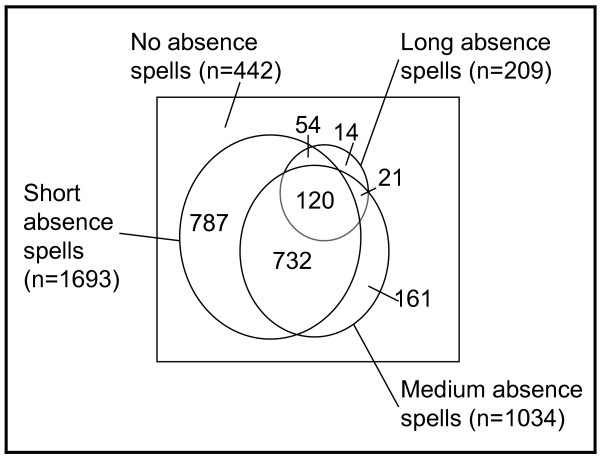
**Distribution of sickness absence spells of different durations (short: 1-3 days, medium: 4-14 days, long: ≥ 15 days)**.

The mean age of the study population was 44 years; nurses were on average the youngest occupational group. Eighty-four percent of the study population were women. Gender was unequally distributed in the occupational groups; the groups of nurses, medical secretaries and physiotherapists consisted of nearly only women and except for the doctors group the other groups consisted mostly of women. Five percent reported poor general health, from 3% of the doctors to 10% among the cleaners/porters group. (Table 1.) Among the 2331 participants, 1889 (81%) had at least one sickness absence spell during the follow-up year. Figure 1 shows the distribution of short, medium and long absence spells. It appears that there were large overlaps.

**Table 1:** Distribution of sickness absence spells and occupational groups by age, gender and self-reported general health

	Total	Age	Women	Fair or poor general health
		mean (SD)	**n (%)**^**1)**^	**n (%)**^**1)**^
**Sickness absence**				
No absence	442	44 (11)	344 (78)	15 (3.4)
Any absence	1889	43 (10)	1610 (85)	106 (5.6)
Any short spells (1-3 days)	1693	43 (10)	1443 (85)	97 (5.7)
Any medium spells (4-14 days)	1034	43 (10)	884 (85)	69 (6.7)
Any long spells (>14 days)	209	45 (10)	190 (91)	24 (12)
"Normal" absence pattern^2)^	970	45 (10)	814 (84)	38 (3.9)
"Abnormal" absence pattern^3)^	919	42 (10)	796 (87)	68 (7.4)
**Occupational group**				
Doctors^4)^	258	45 (10)	109 (42 )	7 (2.7)
Physiotherapists^5)^	294	45 (10)	281 (96 )	24 (8.2)
Nurses	710	41 (10)	681 (96 )	20 (2.8)
Medical secretaries^6)^	328	45 (11)	311 (95)	21 (6.4)
Nursing assistants	491	45 (10)	424 (86)	25 (5.1)
Cleaners/porters^7)^	250	44 (11)	148 (59)	24 (9.6)

1) Percent of total

2) Any absence, but no more than two short, one medium and one long spell, and altogether no more than three spells of any length

3) More than either two short spells, one medium spell or one long spell, or more than three spells of any length

4) Doctors, dentists, psychologists and other academic staff

5) Physiotherapists, midwives, medical laboratory technologists, social workers and alike

6) Medical secretaries, office, IT and administrative workers

7) Cleaning personal, hospital porters, and various assistants

Table 2 shows the sickness absence characteristics in the total sample. Women had more of all types of absences than men. The group of 50-69 years-old had fewer absences of short and medium duration than the other age groups. "Normal" absence increased with age and "abnormal" absence decreased with age. Persons reporting fair or poor health had more of all types of absences, except "normal" absence, than people reporting good or excellent health.

**Table 2:** Sickness absence characteristics among the total sample. By age, gender, general health and occupational group

	Total	Any absence	Any short spells (1-3 days)	Any medium spells (4-14 days)	Any long spells (>14 days)	"Normal" absence **pattern**^**1)**^	"Abnormal" absence **pattern**^**1)**^	Days at risk of a new absence **spell**^**2)**^	Number of absence spells per person-year at risk	Number of absence days in percent of **all working days**^**2)**^**. Group total**
	N	n (%)	n (%)	n (%)	n (%)	n (%)	n (%)	mean (SD)	median	%
**Age**										
18-29	235	191 (81)	175 (74)	106 (45)	22 (9)	76 (32 )	115 (49)	173 (64)	4.00	7.3
30-39	628	512 (82)	469 (75)	299 (48)	41 (7)	246 (39)	266 (42)	182 (62)	3.14	5.6
40-49	715	596 (83)	527 (73)	318 (44)	69 (10)	306 (43)	290 (41)	193 (55)	3.07	6.3
50-69	753	590 (78)	522 (69)	311 (41)	77 (10)	342 (45)	248 (33)	200 (47)	2.05	6.0
**Gender**										
Women	1954	1610 (82)	1443 (74)	884 (45)	190 (10)	814 (42)	796 (41)	190 (55)	3.07	6.4
Men	377	279 (74)	250 (66)	150 (40)	19 (5)	156 (41)	123 (33)	189 (61)	2.06	4.7
**General** **health**										
Fair or poor	121	106 (88)	97 (80)	69 (57)	24 (10)	38 (31)	68 (56)	178 (64)	5.11	11.3
Good, very good or excellent	2177	1757 (81)	1573 (72)	949 (44)	178 (8)	920 (42)	836 (38)	191 (55)	3.03	5.8
**Occupational** **group**^1)^										
Doctors	258	159 (62)	149 (58)	40 (16)	9 (3)	119 (46)	40 (16)	168 (71)	1.06	2.4
Physiotherapists	294	216 (73)	207 (70)	90 (31)	21 (7)	117 (49)	99 (34)	198 (52)	2.06	4.7
Nurses	710	594 (84)	538 (76)	305 (43)	62 (9)	310 (44)	284 (40)	191 (55)	3.07	5.7
Medical secretaries	328	269 (82)	248 (76)	149 (45)	23 (7)	137 (42)	132 (40)	191 (56)	3.01	5.4
Nursing assistants	491	437 (89)	386 (79)	290 (59)	71 (14)	190 (39)	247 (50)	191 (52)	4.05	9.0
Cleaners/ porters	250	214 (86)	165 (66)	160 (64)	23 (9)	97 (39)	117 (47)	197 (50)	3.05	7.2
**Total**	2331	1889 (81)	1693 (73)	1034 (44)	209 (9)	970 (42)	919 (39)	190 (56)	3.04	6.1

1) See table 1

2) Maximum 227 working days in one year.

Fewer in the groups of doctors and physiotherapists had absences (62% and 73%, respectively), than in the other groups (82-89%). Nursing assistants had the highest median number of absence spells, the highest frequencies of short and long spells and of "abnormal" absence, but the lowest of "normal" absence. In contrast, the group of doctors had the lowest median number of absence spells, the fewest absences of short, medium and long duration and the lowest frequency of "abnormal" absence. The cleaners/porters group had much more absence of medium duration (64%) than the other groups.

Table 3 shows results from the final reduced models with adjusted RRs for the incidence of absence spells with the group of doctors as reference. For medium spells, a socioeconomic gradient was obvious with the highest RR being 4.19 (95% CI 2.84-6.19) for the cleaners/porters. For the other outcomes the overall pattern was rather similar except that the RR for the group of cleaners/porters dropped below that of nursing assistants, and for short spells even below that of all other occupational groups. There was also a clear and steep socioeconomic gradient for "abnormal" absence with an OR = 10.5 (95% CI 5.30-20.8) for nursing assistants compared to the group of doctors. The differences were less pronounced for all spells, short spells and "normal" absence. For long spells there were no significant differences between the occupational groups or between any of these and the group of doctors. The confidence intervals were rather wide reflecting that relatively few cases had long spells of sickness absence.

**Table 3:** Effects of occupational group on number and patterns of sickness absence spells.

	All **spells**^**1)**^		Short spells (1-3 **days)**^**2)**^		Medium spells (4-14 **days)**^**3)**^		Long spells (>14 **days)**^**4)**^		"Normal" absence pattern versus no **absence**^**5)**^		"Abnormal" absence pattern versus no **absence**^**6)**^	
	
	Start model	Final model	Start model	Final model	Start model	Final model	Start model	Final model	Start model	Final model	Start model	Final model
Occupational **group**^**7)**^	RR	RR (95% CI)	RR	RR (95% CI)	RR	RR (95% CI)	RR	RR (95% CI)	OR	OR (95% CI)	OR	OR (95% CI)
Doctors	1.00	1.00	1.00	1.00	1.00	1.00	1.00	1.00	1.00	1.00	1.00	1.00
Physio- therapists	1.45	1.43 (1.13-1.83)	1.39	1.36 (1.06-1.74)	1.56	1.52 (1.00-2.32)	1.19	1.03 (0.33-3.21)	1.19	1.29 (0.70-2.37)	2.13	2.27 (1.07-4.83)
Nurses	1.58	1.68 (1.36-2.08)	1.35	1.46 (1.18-1.82)	2.20	2.29 (1.59-3.30)	1.49	1.41 (0.50-3.95)	1.98	2.35 (1.39-3.97)	4.42	5.54 (2.87-10.7)
Medical secretaries	1.64	1.72 (1.37-2.16)	1.34	1.43 (1.14-1.81)	2.65	2.81 (1.92-4.13)	1.10	1.06 (0.35-3.24)	1.71	2.03 (1.12-3.67)	4.83	6.31 (3.06-13.0)
Nursing assistants	1.99	1.95 (1.58-2.41)	1.43	1.47 (1.18-1.82)	3.41	3.34 (2.33-4.80)	2.33	1.89 (0.68-5.28)	2.54	2.90 (1.65-5.09)	9.52	10.5 (5.30-20.8)
Cleaners/ porters	1.52	1.41 (1.10-1.80)	0.83	0.79 (0.60-1.03)	4.30	4.19 (2.84-6.19)	1.63	1.37 (0.45-4.13)	2.09	2.43 (1.25-4.70)	6.60	7.39 (3.33-16.4)
p^8)^	<0.0001	<0.0001	<0.0001	<0.0001	<0.0001	<0.0001	0.10	0.13	0.006	0.001	<0.0001	<0.0001
Mean change of estimates compared to start model		0%		2.2%		0.6%		-11%		15%		17%

Results from multiple Poisson regression analyses (rate ratios (RR) and their 95% confidence intervals (CI)) of all spells, short, medium and long spells, and from multiple logistic regression analysis (odds ratios (OR) and their 95% CI)) of "normal" and "abnormal" absence patterns versus no absence spells.

1) Start model adjusted for age, gender, work-place unit. Final model as start model and for violence, job satisfaction, weekly work hours, being single and general health.

2) Start model adjusted for age, gender, work-place unit, any medium absence, any long absence. Final model as start model and for quality of leadership, social support at work, job satisfaction, weekly work hours, being single and general health.

3) Start model adjusted for age, gender, work-place unit, any short absence, any long absence. Final model as start model and for overall demands, control, job strain, job satisfaction, being single and general health.

4) Start model adjusted for age, gender, work-place unit, any short absence, any medium absence. Final model as start model and for violence and general health.

5) Start model adjusted for age, gender, work-place unit, days at risk. Final model as start model and for overall demands, control, job strain, weekly work hours, overtime work and general health.

6) Start model adjusted for age, gender, work-place unit, days at risk. Final model as start model and for overall demands, control, job strain, weekly work hours and general health.

7) See table 1.

8) Probability of no difference between occupational groups, χ^2^-test.

Table 3 also shows the RR or OR estimates from start models with adjustment for only gender, age, work unit, and effects of other absence spells or days at risk (see section on statistical analyses and footnotes to Table 3), and the mean percentage change of these estimates from the start model to the final model. If the effects of socioeconomic differences were mediated through the covariates in the final model one would expect that risk estimates would change in the direction of unity and that socioeconomic differences in the start model would be reduced [1,14]. However, the risk estimates changed very little. The largest reduction was for long absence spells with an 11% mean reduction of occupational group estimates but the opposite was found for "normal" and "abnormal" absence with a mean increase of 15% and 17%, respectively. The introduction of general health into the models reduced most risk estimates, especially for long absence spells and for "abnormal" absence, especially for the group of cleaners/porters (16% and 17% for the two outcomes, respectively, data not shown). The introduction of work-related psychosocial variables did not reduce the differences in risk-estimates between the occupational groups. On the contrary, they tended to increase the differences, especially for medium and long spells and for "abnormal" absence (data not shown).

Table 4 shows the associations between occupational group and distribution of absence spell durations among participants with any absence. Nursing assistants had the highest proportion of absences of long duration. The doctors group had more absences of short duration and less of medium and long duration than the other groups. Remarkably, among cleaners and porters with any absence, only 22% had only had short spells and 67% had at least had a spell of medium length. For the doctors group the corresponding figures were 73% and 21%.

**Table 4:** Sickness absence characteristics of persons with any sickness absence (n = 1889).

			**Longest absence spell**^**1)**^			Results from logistic regression analyses, final model		
	**Number of** **persons with** **any absence**	**Number of** **absence days** **per year**	**Short** **(1-3 days)**	**Medium** **(4 -14 days)**	**Long** **(>14 days)**	**Medium versus short spells**^**2)**^	**Long versus short spells**^**3)**^	**Long versus medium spells**^**4)**^
**Occupational group**^**5)**^	**n**	**median**	**n (%)**	**n (%)**	**n (%)**	**OR (95% CI)**	**OR (95% CI)**	**OR (95% CI)**

Doctors	159	3	116 (73)	34 (21)	9 (6)	1.00	1.00	1.00
Physio- therapists	216	7	117 (54)	78 (36)	21 (10)	2.24 (1.25-4.03)	1.51 (0.58-3.96)	0.95 (0.32-2.79)
Nurses	594	8	265 (45)	267 (45)	62 (10)	3.24 (1.94-5.40)	2.27 (0.98-5.29)	0.78 (0.29-2.07)
Medical secretaries	269	8	111 (41)	135 (50)	23 (9)	4.49 (2.60-7.78)	2.09 (0.83-5.26)	0.58 (0.20-1.67)
Nursing assistants	437	11	131 (30)	235 (54)	71 (16)	5.84 (3.41-9.99)	4.25 (1.83-9.87)	0.84 (0.31-2.27)
Cleaners/ porters	214	12	47 (22)	144 (67)	23 (11)	11.2 (6.08-20.8)	4.71 (1.82-2.19)	0.72 (0.25-2.07)
Total	1889	9	787 (42)	893 (47)	209 (11)			
p^6)^						<0.0001	0.0003	0.8151
Mean change of estimates compared to start model						6.2%	-11.0%	3.4%

By occupational group. Odds ratios (OR) with 95% confidence intervals (CI) of medium versus short, long versus short and long versus medium spells, adjusted for other significant covariates in multiple logistic regression analysis (see text).

1) Short = only short spells. Medium = any medium but no long spells. Long = any long spells. See figure 1.

2) Adjusted for age, gender, work-place unit, follow-up time, overall demands, control, job strain and general health. Persons with long spells (n = 209) were excluded from the analyses.

3) Adjusted for age, gender, work-place unit, follow-up time,violence and general health. Persons with medium spells (n = 893) were excluded from the analyses

4) Adjusted for age, gender, work-place unit, follow-up time, violence, insecurity at work, duties and general health. Persons with short spells (n = 787) were excluded from the analyses.

5) See table 1.

6) Probability of no difference between occupational groups, χ^2^-test.

For medium versus short spells the ORs increased markedly with decreasing socioeconomic status. The OR for cleaners/porters was 11.2 (95% CI 6.08-20.8) compared to the group of doctors. The pattern was similar but less marked for long versus short spells. The OR for cleaners/porters was 4.71 (95% CI 1.82-2.19) compared to the group of doctors. There were no significant effects of occupational group on long versus medium spells.

Occupational group differences did not change much from a basic model with adjustment for only age, gender, work unit and days at risk to the final model (data not shown). Adjustment for other significant covariates reduced the occupational group ORs by an average of 11% for long versus short spells, and increased slightly for the other comparisons. The effects of introducing general health and work related psychosocial factors into the models followed the same pattern as for the risk estimates of incident absence spells (data not shown).

The proportion of variance explained by random work unit effects was small, approximately 2-7% in all models with individual level covariates (data not shown).

## Discussion

For most of our measures of sickness absence the results showed clear differences between the occupational groups. The group of doctors had fewer absence spells and they were of shorter duration than for the other groups, and the groups of cleaners/porters and nursing assistants had more absence spells and spells of longer duration. The remaining groups were in between.

Our ordering of the occupational groups reflects their socioeconomic status by educational level, positions within the hospital hierarchy and level of wages, except that the group of nurses and the group of physiotherapists should be ranked equal. We did not collapse these two groups because the size of each of them was sufficient to be considered separately in the analyses. The occupational group classification and ordering was based on common knowledge, not on specific personal data except job title.

A socioeconomic gradient was obvious for the incidence of medium spells, "abnormal" absence (table 3), and for the odds of spells of medium and long duration versus spells of short duration (table 4). The incidence of long sickness absence spells was not significantly different for the occupational groups. For the incidence of short spells there was a significant difference between the occupational groups but no obvious socioeconomic gradient. Actually, the lowest socioeconomic group, cleaners and porters, had a lower risk of short spells than the highest socioeconomic group of doctors (table 3). The lack of a socioeconomic gradient in absence spells of 1-3 days may be explained by the increasing proportion of medium versus short spells with decreasing socioeconomic status (table 4). The longer absences in the lower socioeconomic groups could be due to a different pattern of medical causes of sickness absence, to different conditions for returning to work, or to different sickness absence attitudes and behaviours. The lack of a socioeconomic gradient in absence spells of 1-3 days was also found in another study [4]

A socioeconomic gradient in sickness absence is in accordance with results from previous studies [1-8,10-18], but study results are difficult to compare because of different study populations, methods, cultures and legislation, and to different outcome measures Some studies report only results for absence spells of a certain duration, ≥1 day [15], >3 days [12], >7 days [5,8,17], ≥1 week [13], ≥ 14 days [16] and ≥8 weeks [11,14] including persons with none or shorter absence spells in the reference group. Other studies report results for short as well as long absences but with large variations in cut-points, long absences being defined as more than 2 days [6], 3 days [4], 7 days [1,3] and 10 days [7] of absence. The results of our study indicate, that the cut points for absences of different duration may have a considerable impact on the results of a study on socioeconomic effects on sickness absence.

Only a few studies mention the problem that the same person may have several absence spells of different durations. This overlap should be taken into account in the analyses by stratification [29] or statistical adjustment [2], as we did in the present study. However, with a substantial overlap between sickness absences of different duration there is a risk of overadjustment. We therefore reanalysed the final models for short, medium and long absences without adjusting for the effects of other types of absence. The results of these analyses (data not shown) were consistent with the results shown in table 3.

Only a few other studies have examined several different dimensions of sickness absence [30-33]. One study examined sickness absence of '>14 days total', 'mean spell duration >7 days', and '>2 spells of absence' [32]; another studied outcomes defined as '≥3 sick leaves', '> 1 week absence', and '≥1 long spell (>15 days)' [31]. However, these different outcome measures were studied separately. We are not aware of other studies that combined different aspects of sickness absence into a single measure of a distinct absence "pattern". An attractive side of this idea was that it solved the problem of large overlaps between sickness absence spells of different lengths (figure 1). We arbitrarily considered sickness absence as "normal" if a person had no more than two short, one medium and one long absence spell, and no more than three absence spells all together. Any other absence pattern was labelled as "abnormal". By this definition 61% of our population had no absences or a "normal" absence pattern, and 39% had "abnormal" absence. Our first intention was to collapse no absences and "normal" absence to serve as a "normal" reference group to "abnormal" absence. However, as shown in table 2 and 3, even the "normal" absence showed distinct patterns of associations to age, gender, general health and occupational group that were different from those of no absence and "abnormal" absence. Therefore, we report the results for "normal" absence without collapsing this group with the group with no absences. However, our assumptions about a "normal" absence were partly met since the socioeconomic gradient for "normal" absence was much less pronounced than for "abnormal" absence (table 3). There was also an effect of general health on "normal" sickness absence, but much weaker than for "abnormal" absence (data not shown).

We acknowledge that our definition of normal/abnormal sickness absence is based solely on the subjective opinions of the authors. However, our definition was made before analysing the data and we did not explore alternative definitions. Although our a priori assumption that "normal" absence was not associated with socioeconomic status and general health proved to be wrong, we suggest that the approach of defining distinct patterns of sickness absence should be further elaborated using more sophisticated analytical and objective methods in future studies.

We were only able to explain very little of the occupational group differences in sickness absence despite controlling for a large number of potential risk factors, including work time and schedule variables, an extensive set of psychosocial work environment variables, family and personal aspects, and general health. Self-rated general health was a consistent, strong and statistically significant risk factor for all aspects of sickness absence, and was rated poorer with decreasing socioeconomic status (Table 1). These results are in accordance with other studies [5,21,23,34]. However, occupational group differences in sickness absence diminished only a little when general health was controlled for. Thus, in our study, general health only seemed to act as a weak mediator of socioeconomic differences in sickness absence. This is in accordance with some [8,16] but not with other studies [18]. Socioeconomic differences in sickness absence may differ by type of medical disorder[4,10,35]. In Denmark, however, information about medical disorders as causes of sickness absences is neither systematically required nor recorded.

We found only a few significant effects of work related psychosocial factors on sickness absence. Occupational group differences were not explained by these factors. In fact, adjustment for psychosocial factors tended to increase the differences (data not shown). Our results are in accordance with some [14,16] but not with other studies [10,17,18]. In a representative sample of employees in Norway, psychosocial work environment did not explain socioeconomic differences in sickness absence spells of ≥14 days [16]; in a random sample of Danish employees with sickness absence exceeding 8 weeks psychosocial work environment explained very little of socioeconomic differences after adjustment for physical work environment factors [14]. In other studies, psychosocial work environment explained from 24% to 46% of the socioeconomic differences in sickness absence [10,17,18]. Physical working conditions seemed to be a stronger determinant of sickness absence than psychosocial working conditions [14] and a stronger modifier of socioeconomic effects [14]. However, the size of attributable fractions depends on other factors in the model. A better understanding of the causal pathways leading to sickness absence requires repeated measurements of factors of interest at regular intervals.

We have limited our study to sickness absence in the work unit where the participants worked when they filled the questionnaire, and we used maximally one year follow-up on sickness absence. We therefore believe that risk factors recorded at baseline have been rather stable during the observation period. Furthermore, incidence rates were strictly based on days at risk of a new absence spell, excluding all sickness absence days, except for the first day, and all days with absences for other reasons. Another strength of our study is the high response rate which makes it unlikely that non-response bias could seriously distort the pattern of effect estimates and interpretation of study results.

Limitations of the study include its generalizability, being a study of a single large hospital. Also, we would have preferred more information on medical and other reasons for absences, and exact dates on sickness absences and days at risk. Lack of information about specific physical word loads and about life style risk factors is also a shortcoming. However, the effect of life style risk factors on sickness absence may be mediated, at least partly, by their effect on general health which we controlled for in the analyses. Finally, several potential confounders were measured by a single item which is less reliable than a multi-item scale measuring the same construct. However, the lower reliability may be compensated by a larger study population [36].

## Conclusions

We found clear differences in sickness absence between the occupational groups. A strong socioeconomic gradient was found for the incidence of medium spells and "abnormal" absence; and for persons with sickness absences the proportion of medium spells increased and the proportion of short spells decreased with decreasing socioeconomic status. Thus, socioeconomic status was differently related to sickness absence of different duration and pattern. We found no clear explanation for the relations between sickness absence and socioeconomic status. Sickness absence increased with poor general health but general health explained very little of the association between sickness absence and socioeconomic status. Work related factors and personal factors had only sporadic significant effects in this study. However, some of these factors were only measured by a single item.

## List of abbreviations

RR: rate ratio; OR: odds ratio; CI: confidence interval

## Competing interests

The authors declare that they have no competing interests.

## Authors' contributions

TRK participated in the design and analyses of the study and drafted the manuscript. SMJ and SK designed and performed the statistical analyses. SM conceived the study, participated in its design and in the statistical analyses, and helped to draft the manuscript. All authors read and approved the final manuscript.

## Pre-publication history

The pre-publication history for this paper can be accessed here:

http://www.biomedcentral.com/1471-2458/10/643/prepub
